# Rural vs urban residence and experience of discrimination among people with severe mental illnesses in Ethiopia

**DOI:** 10.1186/s12888-019-2345-7

**Published:** 2019-11-05

**Authors:** Sarah Forthal, Abebaw Fekadu, Girmay Medhin, Medhin Selamu, Graham Thornicroft, Charlotte Hanlon

**Affiliations:** 1 Department of Epidemiology, Columbia University, Mailman School of Public Health, New York, NY USA; 2 Centre for Innovative Drug Development and Therapeutic Trials for Africa (CDT-Africa), College of Health Sciences, Addis Ababa University, Addis Ababa, Ethiopia; 3 Department of Psychiatry, School of Medicine, College of Health Sciences, Addis Ababa University, School of Medicine, Addis Ababa, Ethiopia; 4 Department of Global Health & Infection, Brighton and Sussex Medical School, Brighton, UK; 5 King’s College London, Institute of Psychiatry, Psychology and Neuroscience, Department of Psychological Medicine, Centre for Affective Disorders, London, UK; 6 Aklilu Lemma Institute of Pathobiology, Addis Ababa University, Addis Ababa, Ethiopia; 7 King’s College London, Institute of Psychiatry, Psychology and Neuroscience, Health Service and Population Research Department, Centre for Global Mental Health, London, UK

**Keywords:** Global mental health, Mental illness, Discrimination, Stigma, Psychotic disorders, Bipolar disorder, Urbanization

## Abstract

**Background:**

Few studies have addressed mental illness-related discrimination in low-income countries, where the mental health treatment gap is highest. We aimed to evaluate the experience of discrimination among persons with severe mental illnesses (SMI) in Ethiopia, a low-income, rapidly urbanizing African country, and hypothesised that experienced discrimination would be higher among those living in a rural compared to an urban setting.

**Methods:**

The study was a cross-sectional survey of a community-ascertained sample of people with SMI who underwent confirmatory diagnostic interview. Experienced discrimination was measured using the Discrimination and Stigma Scale (DISC-12). Zero-inflated negative binomial regression was used to estimate the effect of place of residence (rural vs. urban) on discrimination, adjusted for potential confounders.

**Results:**

Of the 300 study participants, 63.3% had experienced discrimination in the previous year, most commonly being avoided or shunned because of mental illness (38.5%). Urban residents were significantly more likely to have experienced unfair treatment from friends (χ^2^(1) = 4.80; *p* = 0.028), the police (χ^2^(1) =11.97; *p* = 0.001), in keeping a job (χ^2^(1) = 5.43; *p* = 0.020), and in safety (χ^2^(1) = 5.00; *p* = 0.025), and had a significantly higher DISC-12 score than those living in rural areas (adjusted risk ratio: 1.66; 95% CI: 1.18, 2.33).

**Conclusions:**

Persons with SMI living in urban settings report more experience of discrimination than their rural counterparts, which may reflect a downside of wider social opportunities in urban settings. Initiatives to expand access to mental health care should consider how social exclusion can be overcome in different settings.

## Background

Stigma has been branded “the most basic cultural and moral barrier” to ending the global burden of mental illness [1]. Discrimination is the behavioural consequence of stigma, in which people are treated unfairly due to their condition [2]. Perceived or experienced discrimination can negatively impact upon help-seeking, access to care, recovery, and overall well-being among those with mental illnesses [2]. This may be particularly important in resource-poor settings, where the percentage of people who do not receive necessary mental health care is usually greater than 75% [3]. Furthermore, as the world urbanizes, with low-income nations doing so at the fastest rate [4], there has been increasing interest in better understanding the implications of urban versus rural living on overall mental health and well-being [5].

Nevertheless, there have been very few studies of discrimination in low-resource settings [6, 7] in general, and in sub-Saharan Africa in particular [8, 9]. Existing studies have rarely compared rural and urban populations, despite evidence of high levels of health inequity by residence on the one hand [10] and the prevailing view that more traditional, rural communities may be more tolerant of people with mental health problems [11]. In population-based studies from Ethiopia, both urban [12] and rural [13, 14] residence have been linked with higher levels of stigma, but levels have not been compared in the same study. Rural residence was independently associated with internalised stigma in a study carried out in a tertiary referral centre in the capital city, but this may be explained by selection bias [14]. In this study, we aimed to evaluate the experience of discrimination among persons with severe mental illnesses (SMI) in Ethiopia. Given findings from the Ethiopia Butajira population-based cohort studies showing excess mortality and low remission among those with severe mental illnesses (SMI; psychotic disorders and bipolar disorder) from a predominantly rural setting [15], we hypothesized that, in the Ethiopian setting, rural residence would be associated with higher levels of experienced discrimination in people with SMI.

## Methods

### Study design

The study was a cross-sectional survey of community-ascertained people with SMI in an Ethiopian district as part of the Programme for Improving Mental health carE (PRIME) study [16]. PRIME was a consortium of mental health researchers, the World Health Organization, Ministry of Health representatives and non-governmental organisations in five low and middle-income countries, including Ethiopia. In PRIME, participatory district level mental health care plans were developed in order to evaluate the impact of task-shared mental health care on disability and symptom severity for people with priority disorders [17]. The larger PRIME study includes participants with depression, alcohol use disorder, psychosis, and epilepsy; the current study only includes those with psychosis.

### Setting

The study took place in Sodo District, Gurage Zone, of the Southern Nations, Nationalities and Peoples’ Region (SNNPR), Ethiopia, between December 2014 and July 2015. The district is located 100 km from the capital city, Addis Ababa. Reflecting Ethiopia as a whole, 90% of the district inhabitants live rurally with the main sources of livelihood being farming and animal husbandry. The official language is Amharic. At the time of the study, the district had a population of approximately 165,000 with no mental health specialists. However, through PRIME, a district level mental health care plan was being implemented with the goal of expanding access to integrated primary mental health care [18].

### Participant selection

**Fig. 1:**
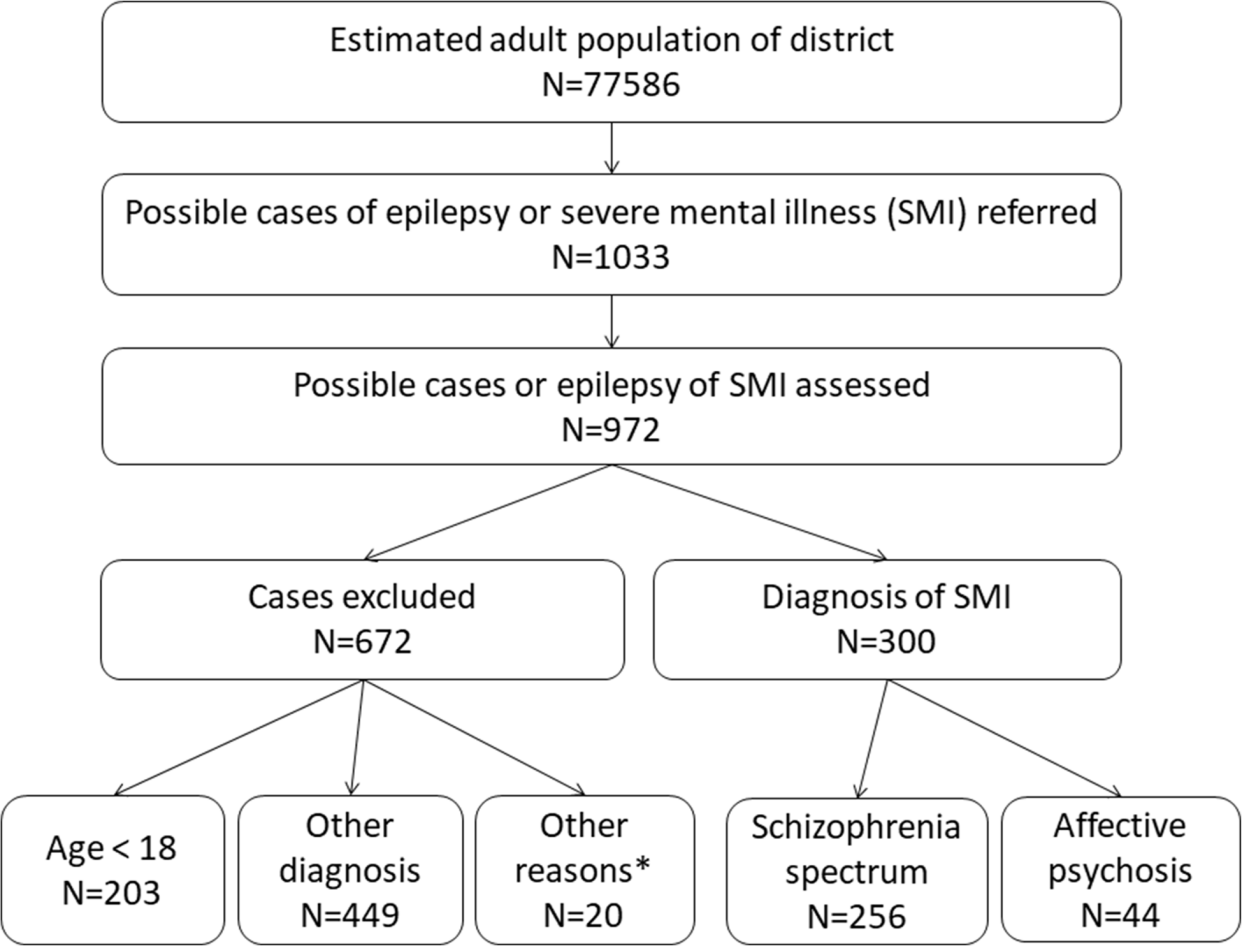
Flowchart of community ascertainment of participants. ^a^Other diagnoses included epilepsy (*n* = 304 and other diagnoses that were not SMI). ^b^Other reasons were: refusal (*n* = 2); language (n = 3); not wanting to transfer from specialist mental health care (*n* = 6); in remission (*n* = 9).

Figure 1 shows a flow chart of the participant recruitment process. Health Extension Workers (HEWs) and community key-informants first identified probable cases of SMI. The key informant method is a sensitive case detection technique previously used in a neighbouring district. HEWs are women with at least a grade 10 education and 1 year of health care training in health promotion and illness prevention activities. HEWs live in the community they serve and visit each household in their catchment area every month and therefore have close community ties [18]. The use of community-based case ascertainment methods allowed us to recruit a more representative sample with minimal selection bias; this is particularly important in a setting such as Sodo District where access to facility-based health care is low. Probable cases of SMI were referred to local PHC services and were then evaluated by nurses or health officers who had been trained in use of the World Health Organization mental health Gap Action Programme Intervention Guide (mhGAP-IG) to diagnose and treat people with psychosis or bipolar disorder [19]. A trained psychiatric nurse confirmed the diagnoses using the semi-structured OPerational CRITeria for research (OPCRIT) interview [20].

People with confirmed SMI and their caregivers were then recruited into the study if they met the following criteria:

- Aged 18 years or older,
- Planning to continue living in the district for the next 12 months,
- Provided informed consent (evaluated by trained psychiatric nurses) or, if lacked capacity to consent, did not refuse and caregiver permission was obtained, and
- Able to understand Amharic.

Those diagnosed with a mental, neurological and substance use (MNS) disorder prior to the study were not excluded.

### Sample size and power calculation

The sample size was powered for the primary objectives of the PRIME study: detection of a 20% reduction in symptom severity after 12 months, with 90% power, two-sided significance level of 5%, and an assumed attrition rate of 20% [21]. The final analytic sample had 300 participants; this was the baseline psychosis cohort for the PRIME study.

### Measures

The primary outcome of the current study was discrimination experienced by people with SMI and the primary exposure was residence. Potential confounders were clinical diagnosis, symptom severity, disability, alcohol use, social support, poverty, age, sex, marital status, and education level. All assessments were administered directly to participants. Most participants had caregivers present during interviews. The caregivers were able to contribute to the responses so that the assessor had the most complete information available to them.

#### Primary outcome: discrimination

Discrimination was assessed by lay interviewers using section 1 of the Discrimination and Stigma Scale (DISC-12), which asks about the frequency of negative experienced discrimination over the past year [22]. Responses are based on a four-point Likert scale ranging from “not at all” to “a lot”. The DISC-12 has been validated [22] and adapted in numerous countries, including Nigeria and Kenya [9]. Responses for two items, ‘unfair treatment in getting welfare benefits or disability pensions’ and ‘unfair treatment in the level of privacy’, were not collected due to lack of local face validity. After conducting exploratory factor analysis with pairwise polychoric correlation on the study dataset, the following two items were found to have low item-factor loading (< 0.3) as well as being endorsed with low frequency (< 5%): ‘Unfair treatment when getting help for physical health problems’ and ‘unfair treatment from mental health professionals’. This left 17 of the original 21 items loading onto one factor, indicating construct validity of the scale. The 17 DISC-12 items were, therefore, summed for a total score.

#### Primary exposure: residence

Residence was self-reported as either urban or rural neighbourhood.

#### Potential confounders

Information on potential confounding variables was obtained from measures administered by [1] psychiatric nurses and [2] lay data collectors.

1. Psychiatric nurse-administered measures

##### Clinical diagnosis

Categorised as affective psychosis (bipolar, schizoaffective, major depressive disorder with psychotic features and postpartum psychosis), or primary psychotic disorder (schizophrenia and other non-affective psychotic disorders) based on the OPCRIT.

##### Symptom severity

Symptom severity was determined by using the Brief Psychiatric Rating Scale - Expanded Version (BPRS-E) translated into Amharic [23]. The BPRS-E is a 24-item tool with seven possible responses ranging from “absent” to “extremely severe”, based on self-reported concerns and clinician observation. The 24 items were summed for a total score. The BPRS-E has been used in Ethiopia previously.

(2) Lay data collector-administered measures

##### Disability

The 36-item World Health Organization Disability Assessment Schedule (WHODAS-2.0) questionnaire measures difficulties performing activities over the last 30 days due to all health problems [24]. The complex scoring method was used to determine a total score, ranging from 0 to 100, where 0 = no disability and 100 = full disability [24]. The WHODAS-2.0 has been validated in a neighbouring district [25].

##### Alcohol use

The Alcohol Use Disorders Identification Test (AUDIT) self-reported version measures alcohol use in the past 3 months [26]. Total score was categorized as either no alcohol use problem (< 8) or hazardous use (≥8).

##### Social support

The Oslo 3-item Social Support Scale (OSSS) asks about ease of getting practical help, number of close acquaintances, and level of concern from others [27]. Responses are categorized as poor support [3–8], intermediate support [9–11], or strong support [12–14]. The OSSS has been previously used in the district.

##### Poverty

Calculated based on indicators used in the 2011 Ethiopia Demographic and Health Survey [28]. Using exploratory factor analysis with maximum likelihood estimation, the following were found to load onto one factor and were summed to form a poverty index: thatched (vs. corrugated iron) roof, non-improved toilet, no separate room for cooking, no electricity, unprotected water source, not possessing a radio or television, or mobile phone. The index was dichotomized at the median [4] to categorise households into higher vs. lower poverty status.

##### Age, sex, marital status, and education level

These variables were self-reported.

### Statistical analysis

Data management and analysis were done using STATA version 15.1 [29]. Categorical variables were summarized by frequency and percent, continuous variables by mean and 95% confidence intervals (CIs). Zero-inflated negative binomial regression models were used to test the relationship between residence and discrimination, including adjustments for the potential confounders listed above. The negative binomial distribution is appropriate for modelling over-dispersed count data. The distribution of discrimination was determined to be zero-inflated upon visual examination.

The potential confounders were determined a priori, as described above, and were included in the adjusted model regardless of statistical significance.

Coefficients are on a log scale; they have been exponentiated and presented as risk ratios for ease of interpretation. The risk ratio represents the increase in total discrimination score for a one-unit increase in explanatory variable.

## Results

### Participant characteristics

A total of 300 people with SMI were included in the analysis. The majority of participants were Orthodox Christians (90.0%), of Gurage ethnicity (94.7%), and resided in rural neighbourhoods (79.9%); 36.9% were unemployed. Primary psychotic disorders (schizophrenia and psychotic disorders) were the most common diagnoses (85.3%) (Table 1).

**Table 1:** Characteristics of persons with SMI included in the analysis

Variable	Rural residence (*N* = 239)	Urban residence (*N* = 60)	Total (*N* = 300)
	N (%)	N (%)	N (%)
Sex			
Male	136 (56.9)	36 (60.0)	172 (57.3)
Female	103 (43.1)	24 (40.0)	128 (42.7)
Education level *(N = 299)*			
No formal education	99 (41.4)	18 (30.0)	118 (39.3)
Formal education	140 (58.6)	42 (70.0)	182 (60.7)
Occupation *(N = 298)*			
Farming	71 (30.0)	5 (8.3)	76 (25.5)
Self-employed	10 (4.2)	6 (10.0)	16 (5.4)
Other employed	24 (10.1)	14 (23.3)	38 (12.8)
House wife	48 (20.3)	10 (16.7)	58 (19.5)
Unemployed	84 (35.4)	25 (41.7)	110 (36.9)
Socioeconomic status *(N = 297)*			
Lower poverty	129 (54.4)	47 (79.7)	177 (59.6)
Higher poverty	108 (45.6)	12 (20.3)	120 (40.4)
Marital status			
Married	88 (36.8)	23 (38.3)	111 (37.0)
Single, divorced or widowed	151 (63.2)	37 (61.7)	189 (63.0)
Religion			
Orthodox Christian	224 (93.7)	45 (75.0)	270 (90.0)
Other	15 (6.3)	15 (25.0)	30 (10.0)
Ethnicity			
Gurage	229 (95.8)	54 (90.0)	284 (94.7)
Other	10 (4.2)	6 (10.0)	16 (5.3)
Diagnosis			
Primary psychotic disorder	208 (87.0)	47 (78.3)	256 (85.3)
Affective psychosis	31 (13.0)	13 (21.7)	44 (14.7)
AUDIT			
No alcohol use problem (< 8)	171 (71.5)	41 (68.3)	213 (71.0)
Hazardous use (≥8)	68 (28.5)	19 (31.7)	87 (29.0)
Oslo Social Support *(N = 298)*			
Poor [3–8]	69 (29.1)	22 (36.7)	91 (30.5)
Intermediate [9–11]	127 (53.6)	24 (40.0)	151 (50.7)
Strong [12–14]	41 (17.3)	14 (23.3)	56 (18.8)
	Mean (95% CI)	Mean (95% CI)	Mean (95% CI)
Age (years)	35.6 (21.8, 49.4)	35.6 (23.8, 47.4)	35.5 (22.1--48.9)
WHODAS 2.0 complex score	44.3 (26.3, 62.3)	40.3 (21.8, 58.8)	43.2 (25.1--61.3)
BPRS-E total score *(N = 294)*	48.9 (33.5, 64.3)	47.4 (27.9, 60.9)	48.5 (32.9--64.1)

*AUDIT* Alcohol Use Disorder Identification Test, *WHODAS* World Health Organization Disability Assessment Schedule, *BPRS-E* Brief Psychiatric Rating Scale-Expanded Version

### Residence and discrimination

Two-thirds of the respondents reported experiencing discrimination (63.3%). There was some variation between individual discrimination items, with 38.3% experiencing being avoided or shunned by those aware of their mental condition, compared to 6.3% experiencing unfair treatment in their religious practices. The most common response to the DISC-12 discrimination questionnaire (‘how many times treated unfairly in the past year’) was “not at all” or “not applicable”, with 36.7% answering “not at all” or “not applicable” to all 17 items (Table 2).

**Table 2:** Responses for individual discrimination items: *treated unfairly in the past year*

Item	Response, N (%)				
	Not at all	A little	Moderately	A lot	Not applicable
Making or keeping friends	210 (70.0)	16 (5.3)	30 (10.0)	35 (11.7)	9 (3.0)
People in your neighborhood	234 (78.0)	17 (5.7)	31 (10.3)	17 (5.7)	1 (0.3)
Dating or intimate relationships	225 (75.0)	12 (4.0)	24 (8.0)	10 (3.3)	29 (9.7)
Housing	266 (88.7)	8 (2.7)	4 (1.3)	19 (6.3)	3 (1.0)
Education	220 (73.3)	5 (1.7)	7 (2.3)	4 (1.3)	64 (21.3)
Marriage or divorce *(N = 299)*	192 (64.2)	12 (4.0)	16 (5.4)	15 (5.0)	64 (21.4)
Family	262 (87.3)	12 (4.0)	7 (2.3)	18 (6.0)	1 (0.3)
Finding a job	233 (77.7)	15 (5.0)	16 (5.3)	16 (5.3)	20 (6.7)
Keeping a job	229 (76.3)	16 (5.3)	8 (2.7)	14 (4.7)	33 (11.0)
Using public transport	261 (87.0)	11 (3.7)	17 (5.7)	8 (2.7)	3 (1.0)
Religious practices	281 (93.7)	7 (2.3)	8 (2.7)	4 (1.3)	0 (0.0)
Social life	254 (84.7)	15 (5.0)	20 (6.7)	7 (2.3)	4 (1.3)
Police	279 (93.0)	6 (2.0)	10 (3.3)	4 (1.3)	1 (0.3)
Physical health treatment^a^	296 (98.7)	2 (0.7)	1 (0.3)	0 (0.0)	1 (0.3)
Mental health staff^a^	298 (99.3)	1 (0.3)	1 (0.3)	0 (0.0)	0 (0.0)
Personal safety and security	241 (80.3)	12 (4.0)	28 (9.3)	19 (6.3)	0 (0.0)
Starting a family or having children *(N = 299)*	219 (73.2)	6 (2.0)	8 (2.7)	7 (2.3)	59 (19.7)
Role as a parent	215 (71.7)	8 (2.7)	9 (3.0)	10 (3.3)	58 (19.3)
Avoided or shunned	185 (61.7)	37 (12.3)	37 (12.3)	41 (13.7)	0 (0.0)

^a^Items were excluded from the total discrimination score and multivariable analysis due to low frequency of endorsement

**Fig 2:**
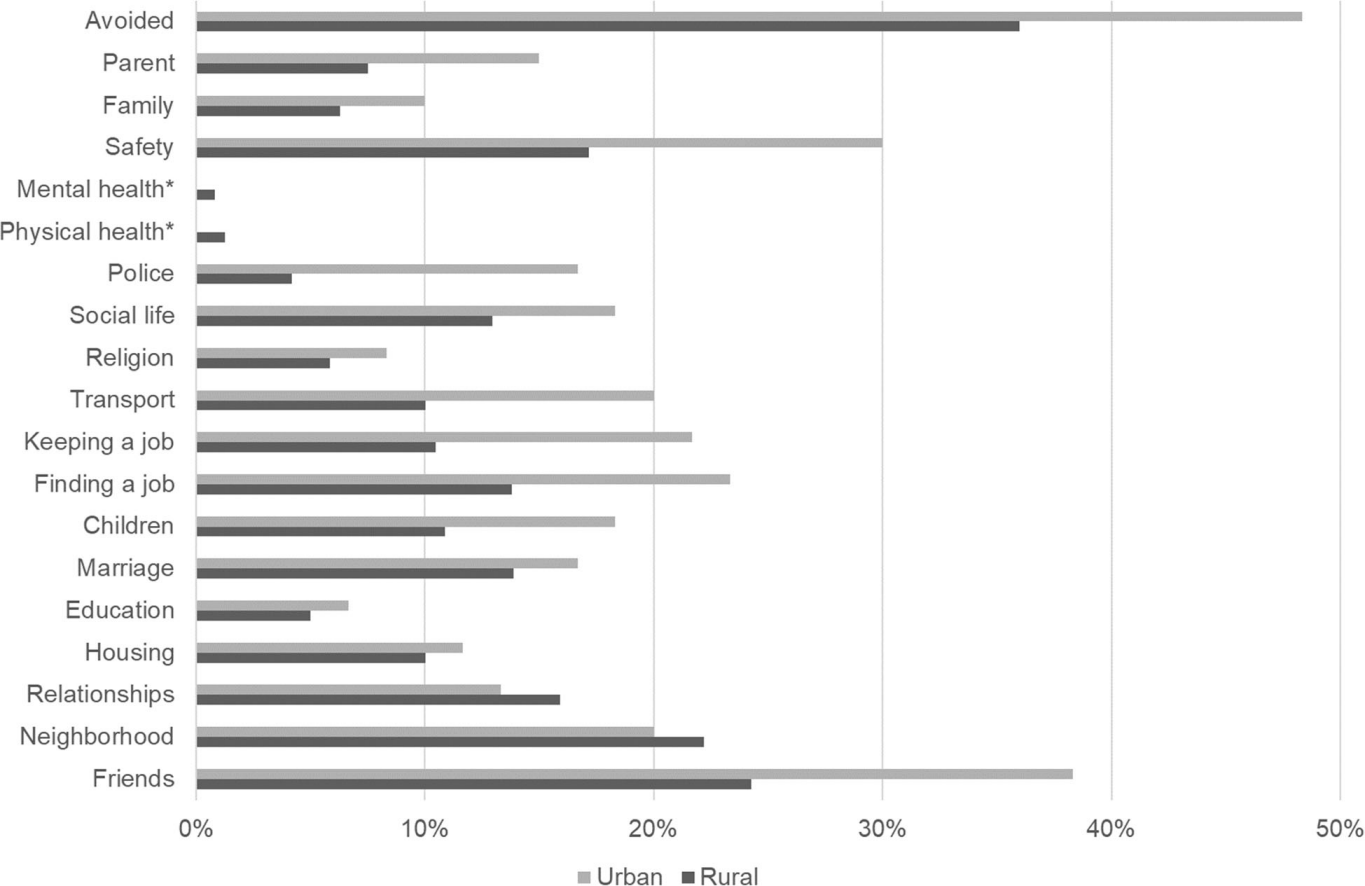
Distribution of respondents reporting any discrimination, by living place

Those from urban neighbourhoods experienced more discrimination on all items except unfair treatment by people in their neighbourhood and in dating or intimate relationships (Fig 2). Urban residents were significantly more likely to have experienced unfair treatment from friends (*χ*^2^(1) =4.80; *p* = 0.028), the police (*χ*^2^(1) =11.97; *p* = 0.001), in keeping a job (*χ*^2^(1) =5.43; *p* = 0.020), and in safety (*χ*^2^(1) =5.00; *p* = 0.025). Approximately half (52.0%) of respondents reported at least “moderate” discrimination on one or more items and 29.9% reported “a lot” of discrimination on one or more items.

### Multivariable analysis

Residence was significantly associated with experienced discrimination: participants living in urban areas experienced 1.66 times the discrimination of those living in rural areas, after adjusting for potential confounders (95% CI: 1.18, 2.33). Female gender (adjusted risk ratio: 1.42; 95% CI: 1.01, 2.00), hazardous alcohol use (adjusted risk ratio: 2.16; 95% CI: 1.51, 3.08), and disability (adjusted risk ratio: 1.02; 95% CI: 1.01, 1.03) were also independently and significantly associated with discrimination in the adjusted model (Table 3).

**Table 3:** Factors associated with discrimination in persons with SMI

Characteristics	Crude Discrimination Multiplier (95% CI)	Adjusted Discrimination Multiplier^a^ (95% CI)
Residence *(N = 299)*		
Urban	**1.51 (1.06, 2.16)**	**1.66 (1.18, 2.33)**
Sex		
Female	0.94 (0.69, 1.27)	**1.42 (1.01, 2.00)**
Education level *(N = 299)*		
Formal education	0.85 (0.62, 1.16)	1.03 (0.75, 1.41)
Socioeconomic status *(N = 297)*		
Higher poverty	1.09 (0.80, 1.49)	1.15 (0.85, 1.54)
Marital status		
Single, divorced, or widowed	0.80 (0.58, 1.09)	0.76 (0.54, 1.07)
Diagnosis		
Primary psychotic disorder	1.00 (0.65, 1.55)	0.85 (0.56, 1.30)
AUDIT		
Hazardous use (≥8)	**1.83 (1.35, 2.48)**	**2.16 (1.51, 3.08)**
Oslo Social Support *(N = 298)*		
Intermediate [9–11]	0.74 (0.53, 1.03)	0.92 (0.67, 1.25)
Strong [12–14]	0.78 (0.48, 1.27)	0.86 (0.55, 1.35)
Age	1.01 (0.99, 1.02)	0.99 (0.98, 1.01)
WHODAS 2.0 complex score	**1.02 (1.01, 1.03)**	**1.02 (1.01, 1.03)**
BPRS-E total score *(N = 294)*	1.01 (1.00, 1.02)	1.00 (0.99, 1.01)
BPRS-E hostility *(N = 294)*	0.87 (0.63, 1.19)	0.92 (0.70, 1.21)
BPRS-E bizarre behavior *(N = 294)*	1.19 (0.86, 1.66)	1.09 (0.79, 1.50)
BPRS-E self-care *(N = 294)*	1.00 (0.72, 1.38)	0.83 (0.61, 1.13)

^a^Adjusted for all factors listed in the table; values in bold are statistically significant

*AUDIT* Alcohol Use Disorder Identification Test, *WHODAS* World Health Organization Disability Assessment Schedule, *BPRS-E* Brief Psychiatric Rating Scale-Expanded Version

## Discussion

People with SMI residing in urban neighbourhoods reported experiencing 1.66 times the discrimination compared to those residing in rural neighbourhoods, after adjusting for potential confounders. This finding is in agreement with a study from a neighbouring district which found that perceived stigma was more common among urban family members of those with SMI [12]. Our results can be read in the context of the landmark international studies by the World Health Organization [11, 30], which found that people with schizophrenia in “developing” countries had more favourable prognoses than those in “developed” countries. It is often considered that this may be due to a greater tolerance towards mental illness in low-income countries [31]; however, our results suggest that levels of tolerance vary by living place within Ethiopia. Although people with SMI in the population-based Ethiopian Butajira cohort did not have superior outcomes compared to people with SMI living in high-income countries, there was no difference in outcomes by residence [15, 32]. This is somewhat unexpected, as most health states have a worse outcome in rural settings due to a constellation of factors related to poor access to health care, lower health (and mental health) literacy and poorer socio-economic status [10]. It is possible that the higher rates of discrimination observed among urban residents compared to rural residents offset the health benefits of urban living.

The difference between urban and rural levels of discrimination might be explained by the nation’s recent rapid growth and urbanization. Ethiopia has the fastest growing economy in sub-Saharan Africa and the proportion of the population living in urban areas is on track to triple from its 2012 levels by 2028 [33]. The World Bank reports that job creation, infrastructure, and housing have not been adequate in handling the influx of migrants, leading to greater inequality of income and quality of life [33]. Thus, it may be more difficult for people with mental illness to find meaningful work and integrate into the daily life of urban areas. This is particularly consequential as the decision to migrate is often based on a promise of economic opportunity, yet, facing higher levels of discrimination in their new residences may actually lead to reduced opportunities. It is sometimes assumed that individuals in rural communities have stronger social and familial support networks, and that this may protect against discrimination [31]. However, in our sample, the strongest social support was reported by urban residents. Further, our analysis controlled for social support, so the pattern of more urban discrimination persisted despite any social support reported by participants.

Over one-third of the participants reported no discrimination on any items. This conflicts with findings of consistently high levels of stigma in Ethiopia [12, 13, 34]. One potential reason for this finding is that respondents may not believe they are being treated unfairly and that their condition justifies differential treatment. High levels of “not applicable” responses in our data may be an indication of this. For instance, 19.7% of respondents answered “not applicable” to being discriminated against in “starting a family or having children”. While some of these responses may be due to having no interest in starting a family, being too old, or already having a family, it is also possible that they did not feel as though starting a family was an appropriate endeavour for them. This concept can be described as self-stigma, which occurs when stigmatizing attitudes are internalized by the recipient [35]. In a hospital-based study from Addis Ababa, Ethiopia’s capital, self-stigma among people with schizophrenia was ubiquitous, and significantly higher amongst rural residents [14]. However, this study had a primarily urban, wealthier, and better educated population, and may not be fully comparable to our study population. Self-stigma may have contributed to both the low levels of overall reported discrimination and differences between rural and urban residents, but more research is needed to better understand the relationship between self-stigma and reports of discrimination in this setting.

Strengths of the study include the community-based case ascertainment (which reduced the risk of selection bias that is present in facility-based studies), confirmatory diagnostic interviews performed by trained mental health specialists and clinical assessments of symptom severity. However, there were study limitations. There is evidence that the construct and manifestations of discrimination varies between countries [2], and the DISC-12 was not specifically developed for the Ethiopian setting [22]. Social desirability may have affected the willingness of participants to attribute their experiences to discrimination. Bias in the discrimination questions could partially explain the difference between urban and rural discrimination. Certain questions, especially those regarding job-related discrimination, may be less relevant to rural residents, where formal employment is less common. Discrimination in the neighbourhood and in relationships were more common among rural residents, which could be related to less anonymity in these settings. Another limitation of the study is that we did not record whether the person with SMI responded to the DISC-12 questions or whether the caregiver provided proxy responses. In a follow-up wave of data collection for the same sample, 29.4% of responses were from the caregiver alone. Applying this percentage to the baseline dataset (the focus of this paper), we found that there was no significant difference in total DISC-12 scores where the person with SMI responded compared to when the caregiver responses (z = 1.412; *p* = 0.158). Care is also needed when generalizing these results to other Ethiopian populations, as what is considered urban might differ between predominantly rural districts like Sodo, and the capital city of Addis Ababa, for example. Although contact coverage for PRIME’s district mental health care plan was high (81.7%), participants from rural areas were significantly less likely to attend the health centre than their urban counterparts and therefore less likely to be included in this sample [36]; however, non-attendees had lower mean disability scores indicating that they were less severely unwell and likely to be at lower rather than higher risk of discrimination. Lastly, the data are cross-sectional and therefore we cannot make any conclusions about the directionality of the relationship between living place and experienced discrimination.

There is some evidence that social contact and mass educational campaigns can reduce stigmatizing attitudes among the general public [2, 6]. It may be that better access to treatment can help reduce experienced discrimination among people with mental illnesses, though it is unclear to what extent this needs to be augmented with other interventions. In Kenya, a comparable low-treatment setting, the implementation of mhGAP-IG led to a significant decrease in discrimination experienced by mental health service users after six months [9]. Similarly in a trial of community-based care in India, a significant reduction in discrimination after 12 months of treatment was observed [37]. We can speculate that these results are due to effective treatments reducing disability and consequentially improving participants’ abilities to participate more fully in social life, but this is an area that should be explored further. In the PRIME study, where the district mental health care plan relies on existing cadres of staff and has no formal anti-stigma or anti-discrimination intervention, it will be possible to evaluate where a scalable, predominantly facility-based model of care can impact upon discrimination.

## Conclusion

Among persons with SMI in Sodo district, urban residence was associated with greater experienced discrimination. Overall, the reported levels of experienced discrimination were low considering the consistently high levels of stigma reported in the current literature. There may be distinct aspects of urban living in low-income countries, such as inadequate infrastructure and job opportunities, that should be further investigated as potential risk factors for discrimination. Policymakers seeking to expand access to mental health care should be aware of these distinct experiences and consider how social exclusion can be overcome indifferent settings.

## Data Availability

The datasets used and/or analysed during the current study are available from the corresponding author on reasonable request. The PRIME datasets will be made publicly available during 2019 (via www.prime.uct.ac.za).

## Abbreviations

AUDIT: Alcohol Use Disorders Identification Test
BPRS-E: Brief Psychiatric Rating Scale - Expanded Version
DISC-12: Discrimination and Stigma Scale
HEWs: Health Extension Workers
mhGAP-IG: mental health Gap Action Programme Intervention Guide
MNS: mental, neurological and substance use
OPCRIT: OPerational CRITeria
OSSS: Oslo 3-item Social Support Scale
PRIME: Programme for Improving Mental health carE
SMI: severe mental illnesses
SNNPR: Southern Nations, Nationalities and Peoples’ Region
WHODAS-2.0: World Health Organization Disability Assessment Schedule
